# Crust of Bread

**Published:** 1863-11

**Authors:** 


Crust of Bread.—M. Barral has presented to the Academy of Sciences some remarks of much interest concerning the crust of bread and the gluten contained in it. He had recently shown that, when equally dried, the crust of bread is more highly azotized than the crumb; and he also showed that the crust was more soluble than the crumb in water. M. Payen has, it is true, previously pointed out this greater solubility of the crust, and had ascribed it to the conversion of the starch into dextrine during the baking. But M. Barral's experiments show another important fact. "If," he says, "we exhaust with water an equal quantity of dry crumb and dry crust of bread, we find that the soluble portion of the crust consists of from 7 to 8 per cent. of nitrogen, whilst the soluble portion of the crumb contains only from 2 to 3 per cent. The greater solubility of the crust. consequently, depends upon the transformation which its gluten has undergone under the direct action of the 200° to 220° heat of the oven. The soluble portion of the crust is more highly azotized than the juice of meat." M. Barral added, that he was still engaged with his experiments, which he hoped would throw some new light on panification.—*British Med. Journal*, July 11, 1863.

